# An Unusual Presentation of Vertigo: Is Head Titubation the Key to Diagnosis?

**DOI:** 10.1155/2009/358019

**Published:** 2009-02-15

**Authors:** O. Judd, M. Medcalf

**Affiliations:** Department of Otolaryngology, Derriford Hospital, Plymouth PL6 8DH, UK

## Abstract

*Objective*. Discuss complex interplay of pathophysiological effects of cerebellar space occupying lesions on the vestibular pathway. Discuss challenges of diagnosis and referral along with differential and final diagnosis of unusual presentation. *Case Report*. We describe the case of a patient with vertiginous symptoms complicated by neurological features, namely, head titubation and tremor. The patient also had signs of oscillopsia and possible impairment of the vestibulo-ocular reflex. The resulting symptom and sign complex made for a difficult diagnosis, as the interplay of the pathophysiology of these signs, were unusual. *Conclusion*. The discussion has revealed that the cerebellar lesions themselves may have simultaneously caused head tremor and an inability for the vestibulo-ocular reflex to compensate, resulting in vertigo. However, whether the vertigo was a result of an oscillopsia, nystagmus, or central cause, the referral route should initially be via a general physician to rule out such a life threatening cause as a tumour.

## 1. Introduction

A presentation to hospital with vertigo or dizziness is common [1, 2]. The usual reason is disabling dizziness leading to nausea, vomiting, and often prostration or inability to mobilise. This may represent central or peripheral vestibular disease or nonvestibular dysequilibrium [3]. Patients with these symptoms are challenging with difficulty determining which specialty to refer to. Otolaryngologists have expertise in this area; however, patients may be more appropriately referred to neurologists or general physicians in the first instance. We discuss the close interplay of pathophysiology relating to vertigo, nystagmus, oscillopsia, and the vestibulo-ocular reflex and its modulation by head titubation.

Vertigo (Type I dizziness) is a multisensory syndrome. It may be induced by stimulation of the intact sensorimotor system or by dysfunction of any of the stabilising sensory systems. Vertigo can also occur with nonvesibular disease.

General sensations of dizziness, in contrast, are often of nonvestibular aetiology. Whether this be an impending faint (Type II), dysequilibrium (Type III), or vague lightheadedness (Type IV), it may be due to nonvestibular causes. These can include cerebrovascular disease, multiple sclerosis, CNS tumours, migraine, or epilepsy [3].

## 2. Case Report

A sixty-four-year-old lady presented to her general practitioner (GP) with acute onset of unsteadiness and dizziness associated with movement. She visited her doctor with a complaint of feeling dizzy and faint but had a blood pressure of 113/70 mmHg. She had no abnormalities on general, vestibular, neurological, and otological examinations, including no nystagmus. The dizziness was described as repeated attacks of a sensation of internal moving; that is the patient was “whirling”, but the room was still. The attacks lasted for a few hours at a time. Her symptoms worsened over the following week, disturbing her daily activity and leading to nausea, vomiting, and prostration. The GP had noted that she had irregular and erratic eye movement and had developed a postural drop in her blood pressure, from 113/70 mmHg to 97/63 mmHg on standing.

A week later she was permanently dizzy and nauseous and had noticed some difficulty in pronunciation of words. The dizziness was now described as a constant sensation that she was turning and tilting over, when she knew she was upright, and the room was still. This was noted during both still and on movement. There had also been the occasional sensation that the room was spinning around, although these were the only fleeting. She had no visual disturbance, hearing problems, weakness or numbness, and her neurological examination was normal. There was no nystagmus, and fundoscopy was normal. Blood tests were also normal. She was treated with antiemetics including prochlorperazine; with a presumed diagnosis of vestibular failure, and her GP referred her to the otolaryngology services for further treatment and to investigate for an organic cause.

On admission, she was clearly unwell and complained of a mild bilateral tinnitus. She had no headache, neck stiffness, or limb weakness. On examination, she was noted to have a subtle head nodding tremor or titubation. This was associated with a low-frequency tremor of the upper limbs, which appeared to increase on intention. She had erratic eye movements and it was very difficult to elicit any nystagmus, even with Frenzel glasses, whether spontaneous or positional. This was due to the difficulty in keeping her eyes open secondary to overwhelming nausea. Her symptoms of nausea improved on closing her eyes. The patient found that it very difficult to keep her eyes still and to focus on a stationary object and she was unable to smoothly pursue a moving target. There was clearly a fixation instability and it appeared to be in the form of conjugate involuntary saccadic oscillations. It was concluded that this was characteristic of opsoclonus. There was, however, a fleeting vertical nystagmus on upgaze, which may have indicated a gaze-evoked cause, but with no obvious horizontal element. Examination of the ears with otoscopy was normal. A Halmagyi-Curthoys head thrust test was attempted, but proved difficult to interpret due to the stimulation of vomiting. It was noted, however, that the patient was unable to fix on a stationary object during head rotation indicating a probable suppression of the vestibulo-ocular reflex (VOR). Due to the significant nausea and vomiting, a caloric canal test was not performed.

She had subtle cerebellar signs on subsequent examination, including dysmetria and dysdiadocokinesis. Romberg's test was negative. Dix-Halpike test was symptomatically positive, with vomiting but with no rotatory nystagmus and no positional dependence. Her gait was not tested, as she was too unsteady. She was given intravenous fluids and steroids and treated symptomatically for the nausea and vomiting.

**Figure 1:**
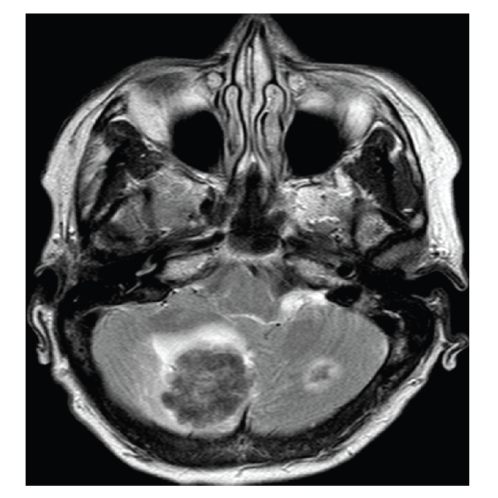
MRI of brain. Note space occupying lesions to cerebellum.

It was postulated that there was a central cause for her symptoms, due to the combination of cerebellar signs, fixation instability/opsoclonus, possible upbeat nystagmus, and VOR suppression. Therefore, a magnetic resonance scan (MRI) of the brain was ordered. This revealed a large space-occupying lesion in the right lobe of the cerebellum, with a smaller lesion in the left lobe (Figure 1). The patient was referred directly to the neurosurgeons who performed a suboccipital craniectomy and excision of the cerebellar tumour. The histology of the lesion was metastatic adenocarcinoma.

## 3. Discussion

As a presentation to otolaryngology, the history of this case is not unusual. Dizziness is experienced by 20% of the working population [2]. In primary care, it accounts for 13% of patients, but only 50% of those referred to otolaryngology services have an otological cause for their symptoms [4]. Other causes may include life threatening conditions such as cerebrovascular events. It can be argued, therefore, that patients presenting with these symptoms should be referred to general physicians in the first instance, to rule out serious nonotological causes.

The anamnesis of dizzy symptoms is especially important to elicit in detail in order to make informed decisions on referral route. Vertigo indicates a sensation of false movement (generally described like a rotation), but rarely, the patient can describe it like a sensation of tilt [5]. In our case, the patient described both rotational and tilting sensations, whilst stationary and on movement. Most importantly was the constant sensation that she was turning but that the room was still. This specific symptom, and its duration, indicates an increased risk of central origin [6].

With peripheral vestibular causes of vertigo, the sensation tends to be of the outside world spinning. This may be a result of oscillopsia, an optical illusion of oscillation sometimes as a direct result of nystagmus [7]. Oscillopsia is common in bilateral labyrinthine abnormality, but the presence of vertical oscillopsia indicates a vertical nystagmus, which is indicative of a central lesion [8]. In this case, spontaneous nystagmus, albeit fleeting, was vertical nystagmus and this is particularly specific to central lesions rather than peripheral vestibular lesions, and is a reliable indicator [8]. Damage to the ventral tegmental tract, originating in the superior vestibular nucleus, leads to upward vestibular signals to the third nerve nucleus and so upbeat nystagmus (UBN) [9]. UBN is also indicative of cerebellar vermis lesions [8]. Therefore, a vertical nystagmus should give high index of suspicion for a central cause. Spontaneous gaze-evoked nystagmus is also indicative of cerebellar lesions.

The general inability to stabilise the eye, resulting in the erratic eye movement and opsoclonus seen may also indicate central pathology. The cerebellum controls various types of image-stabilising reflexes. Particular regions involved are the vermis, flocculus, and paraflocculus, which are responsible for coordinating smooth pursuit and adaptation of the vestibulo-ocular reflex (VOR) [10]. These control mechanisms are essential to holding the eye steady for fixation, both immediately after saccades and in eccentric positions of gaze, particularly important when attempting to track a moving target. The lesions in the cerebellum may well have impaired these important mechanisms in this patient, leading to opsoclonus and an inability to fixate. It is also known that an impairment of adaptation of the VOR, to compensate for movement, may lead to oscillopsia [11]. Although the lesions seen on MRI in this case are not in the vermis, surrounding oedema and paraneoplastic effects may be responsible for an impairment of vermis function.

The general difficulty in diagnosis, detailed neurotologic examination, and testing in this case may have been influenced by the use of prochlorperazine. This is a phenothiazine antipsychotic agent which is very commonly used as a vestibular sedative for nausea due to vertigo. It is widely known, however, although affording symptomatic relief for the patient, this drug can result in a delay to vestibular compensation and influence diagnostic testing. It is also a well-known cause for oscillopsia.

The most interesting sign in this case was head titubation. This is defined as nodding head tremor with a frequency of 3 to 4 Hz and may be seen in midline cerebellar disease [12, 13]. Titubation can occur in isolation or combined with a postural tremor elsewhere, especially in the arms. This can be seen in patients with essential tremor or dystonic tremor, associated with posterior fossa disorders [13]. Essential tremor, such as this, is closely related to the oculomotor deficits described above. These include deficits of pursuit initiation and suppression of the VOR. It has been shown that there is a strong correlation with the intensity of intention tremor and the oculomotor impairment and may indicate an impairment of the caudal cerebellar vermis [14]. There is the potential, however, that the tremor and head titubation may have been an extrapyramidal akathisia as a result of prochlorperazine use.

The closely related interplay of the mechanisms involved in this patient's symptoms are difficult to sequence. The cerebellar lesions are the only true evidence of a causal element to this case. However, it remains to be understood how these lesions had brought about the specific symptom complex identified. Was the vertigo a direct result of the lesion or was it secondary to an oscillopsia generated by nystagmus, or due to failure of the VOR? Was the oscillopsia due to the head titubation? Was UBN or gaze-evoked nystagmus present, or was it just a brief recognisable pattern in the otherwise erratic oculomotor impairment or opsoclonus, or secondary to loss of adaptation of the VOR?

Was the head titubation the key? Oscillopsia occurs when there is failure of the VOR to adapt and compensate for head movements. Cerebellar lesions may impair the normal cerebellar adaptive mechanism for the VOR and so impair its ability to compensate for movement of the head. Simultaneously, a cerebellar lesion may also lead to an essential tremor, manifesting as head titubation and upper limb tremor. Many of the signs seen in this case, such as the impaired VOR, the titubation, opsoclonus, and dysarthria, are classically caused by cerebellar vermal lesions. However, the lesions seen on MRI are in the deeper hemispheres. One may, therefore, suggest that surrounding oedema and local compression have led to vermal damage. It can be seen in Figure 1 that there is a degree of cerebellar midline shift.

One may conclude that the lesions *themselves* may have simultaneously caused a tremor and an inability for the VOR to compensate for it. This may have resulted in an oscillopsia, manifesting as disabling vertigo. Oscillopsia due to an impaired VOR has been shown to be more likely than oscillopsia due to a titubation [15]. Also, the two cerebellar lesions may have had the two separate effects of tremor and impaired VOR.

The differentiation between central and peripheral causes of vertigo can be perplexing and often require the expertise of a specialist neurotologist. Many signs and tests, such as saccadic pursuit, gaze-evoked nystagmus, and the Halmagyi-Curthoys test, have recently been shown to be unreliable in distinguishing peripheral from central causes; therefore, an expert opinion may be required [16]. However, whether the vertigo in this case was a result of an oscillopsia, nystagmus, or central cause, the referral route should initially be via a general physician to rule out such life threatening causes as central tumours.

## 4. Conclusions

1. Suspicion of a central cause for vertigo on examination should prompt urgent imaging of the central nervous system.
2. A vertical nystagmus should give high index of suspicion for a central cause.
3. Central vestibular lesions may present with a wide variety of symptoms and signs which may include impaired VOR, head titubation and tremor, opsoclonus, and oscillopsia.
4. Initial referral of patients with disabling vertigo should be via general physicians.
